# The Value of Technology to Support Dyadic Caregiving for Individuals Living With Heart Failure: Qualitative Descriptive Study

**DOI:** 10.2196/40108

**Published:** 2022-09-07

**Authors:** Noor El-Dassouki, Kaylen Pfisterer, Camila Benmessaoud, Karen Young, Kelly Ge, Raima Lohani, Ashish Saragadam, Quynh Pham

**Affiliations:** 1 Centre for Global eHealth Innovation Techna Institute University Health Network Toronto, ON Canada; 2 Institute of Health Policy, Management and Evaluation Dalla Lana School of Public Health University of Toronto Toronto, ON Canada; 3 Health Sciences Faculty of Health University of Waterloo Waterloo, ON Canada; 4 Telfer School of Management University of Ottawa Ottawa, ON Canada

**Keywords:** heart failure, digital therapeutics, remote patient management, caregiving, dyadic management

## Abstract

**Background:**

The demand for health services to meet the chronic health needs of the aging population is significant and remains unmet because of the limited supply of clinical resources. Specifically, in managing heart failure (HF), digital health sought to address this gap during the COVID-19 pandemic but highlighted an access issue for those who could not use technology-mediated health care services without the support of their informal caregivers (ICs). The complexity of managing HF symptoms and recurrent exacerbations requires many patients to comanage their illness with their ICs in a care dyad, working together to optimize patient outcomes and health-related quality of life. However, most HF programs have missed the opportunity to consider the dyadic perspective despite interdependencies on HF outcomes.

**Objective:**

This study aims to characterize the value of technology in supporting caregiving for individuals living with HF.

**Methods:**

Motivated by an observed unique pattern of engagement in patients enrolled in our Medly HF management program at the Peter Munk Cardiac Centre in Toronto, Canada, we conducted 20 semistructured interviews with a convenience sample of ICs. All interviews were analyzed using the iterative refinement of a codeveloped codebook. The team maintained reflexivity journals to reflect the impact of their positionality on their coding. Themes were first derived deductively using HF typologies (patient-oriented dyads, caregiver-oriented dyads, and collaboratively oriented dyads) and then inductively refined and recategorized based on concepts from the van Houtven et al framework.

**Results:**

We believe that there is a need to formally and intentionally expand HF technologies to include dyadic needs and goals. We suggest defining 3 opportunities in which value can be added to technological design. First, identify how technology may be leveraged to increase psychological bandwidth by reducing uncertainty and providing peace of mind. We found that actionable feedback was highly desired by both partners. Second, develop technology that can serve as a member of the dyad’s support system. In our experience, automated prompts for patients to take measurements can mimic the support typically provided by ICs and ease their workload. Third, consider how technology can mitigate the dyad’s clinical knowledge requirements and learning curve. Our approach includes real-time actionable feedback paired with a human-in-the-loop, nurse-led model of care.

**Conclusions:**

Our findings identified a need to focus on improving the dyadic experience as a whole by building IC functionality into digital health self-management interventions. Through a shared model of care that supports the role of the patient in their own HF management, includes ICs to expand and enhance the patient’s capacity to care, and acknowledges the need of ICs to care for themselves, we anticipate improved outcomes for both partners.

## Introduction

### Background

The demand for health services to meet the chronic health needs of the aging population is significant but remains unmet because of the limited supply of clinical resources. Although the pandemic-induced scale and spread of digital health sought to address this gap and widen access to care [1], it also highlighted a portion of the population who could not use or benefit from technology-mediated health care services without the support of their informal caregivers (ICs). Defined as individuals who provide unpaid care and assistance to friends or family members because of a health condition, 2.7 million Canadians aged ≥45 years identify as ICs and provide approximately 75% of home care services to support aging in place [1,2]. In this context, we use the general term ICs to refer to either primary or secondary ICs to account for the intricacies of family-oriented care and the ecosystem that the broader circle of care provides within the context of a patient’s care community.

Within digital health and the broader health care system, having support from an IC is particularly important for individuals living with heart failure (HF). As the most complex and costly chronic condition to manage in health care, HF is the single most common reason for hospital admissions and readmissions in Canada [3]. As a result, patients require constant care, largely because of the complexity of HF symptom management and recurring exacerbations [4,5]. This leads many individuals living with HF to comanage their illness with their ICs in a dyadic arrangement, with dyads working together to optimize patient outcomes and health-related quality of life.

Although substantial work has been conducted on patient self-care interventions, most HF programs have missed the opportunity to consider the broader perspective of the dyad [6,7]. As each member of the dyad influences the behavior and well-being of the other, integrating dyadic considerations into the design of HF interventions has been shown to enhance patient health outcomes [6-11]. Most existing HF interventions geared toward dyads have emphasized peer support and mentoring programs, patient education programs, face-to-face education and visits, brochures, and voice calls [7,12]. Although largely unexplored, we anticipate technology to be well positioned to help support dyads to (1) perform several complex tasks associated with the daily demands of HF, such as planning appointments and managing medications; (2) receive real-time feedback as a supplementary support system; and (3) access tailored educational resources to address the high degree of caregiving uncertainty experienced because of insufficient knowledge and training.

We were motivated to pursue this research as our Medly clinical and implementation team observed a unique pattern of engagement in certain patients enrolled in our Medly HF management program at the Peter Munk Cardiac Centre in Toronto, Canada [13]. Patients within the program use the Medly platform as the standard of care to capture daily health measures, including weight, blood pressure, heart rate, and HF-related symptoms. The embedded Medly algorithm immediately analyzes the entered readings against set personalized thresholds and provides the patient with instant feedback and instructions. The algorithm also alerts the patient’s care team on the Medly dashboard for further assessment and triage [14]. Owing to the unacceptably high risk to patients living with HF coming into the hospital at the height of the pandemic, cardiologists in the program began enrolling patients into Medly who would typically not be eligible for the program. The team observed that this new cohort of patients had low digital health literacy and English language proficiency but could be enrolled as they were onboarded with an IC who had agreed to use Medly on their behalf. This off-label dyadic use of an HF digital therapeutic (DTx) allowed us to further explore how we could provide better support for dyads to engage in effective shared care through technology.

### Objective

This study sought to characterize how technology, specifically DTx, might support ICs engaged in the dyadic management of HF to optimize their caregiving practices and improve shared outcomes. We define DTx as the “evidence-based, clinically evaluated software [used] to treat, manage, and prevent a broad spectrum of diseases and disorders” [15]. Specifically, we sought to answer two research questions: (1) what are the challenges of caregiving for an individual living with HF, and (2) how can technology support the dyadic management of HF to enable improved outcomes for both partners?

## Methods

### Conceptual Frameworks

To structure our research strategy and approach, we began by conducting an environmental scan of the existing literature on the dyadic management of HF. We also sought a framework that could help examine the relationship between dyad type and the value of technology in HF management. Buck et al [16,17] conceptualized an HF care dyadic typology by integrating and applying 2 theoretical models, the actor-partner interdependence model and interdependence theory, to the dyadic management of HF. They described 4 categories of dyadic relationships, which we collapsed into 3 to align with our approach: patient-oriented dyads, caregiver-oriented dyads, and collaboratively oriented dyads. In patient-oriented and caregiver-oriented dyads, the person living with HF or the IC takes on most of the self-care responsibilities of the patient living with HF. Conversely, in collaboratively oriented dyads, both the patient and IC have joint involvement in HF self-care [16]. This framework allowed us to strengthen the theoretical basis of our work, add to the evidence base in the validation of this model, and advance our understanding of the role that dyad type plays in the use of technology in HF management. We continually returned to this framework during the course of data collection and analysis, reflecting on the ways in which our data were bounded by the typology and the use of the dyadic relationship as the main frame of analysis.

Upon completing the initial analysis of our data using the typologies outlined by Buck et al [16,17], we sought an organizing framework to further consider and optimize future evaluation efforts. The van Houtven et al [18] framework considers how the baseline characteristics of the patient-caregiver dyad influence the way an intervention is designed and how the caregiver engages with it. The efficacy of an intervention is then evaluated based on the changes it contributes to (1) caregiver activities and (2) caregiver and patient outcomes, which include psychological and physical health, health care use, and financial status. Although this study aimed to create rather than evaluate an intervention, we felt that the work by van Houtven et al [18] provided a strong, standardized framework for determining which factors need to be considered when designing an effective intervention that optimizes patient and caregiver outcomes.

### Study Design

#### Overview

This qualitative descriptive study was conducted at the University Health Network (UHN) between April 2021 and March 2022 during the COVID-19 pandemic. All recruitment and data collection activities were conducted virtually. Data collection was conducted by authors NE and CB, and the data analysis team comprised research staff (NE and CB) and research students (KY and KG).

#### Positionality of the Data Analysis Team

The term positionality refers to the ways in which individuals identify and relate to various social dimensions (ie, gender, race, and ethnicity) [19]. A key element of high-quality qualitative research is sincerity, achieved through self-reflexivity and transparency on the part of the researcher [20]. Being able to openly recognize the researcher’s positionality, experiences, and assumptions and center the active role that the researcher plays in the interpretation of data is a central tenet of reflexive thematic analysis [21]. In alignment with the related concept of researcher subjectivity as a valuable resource, the authors maintained a reflexivity journal and described their social positionalities to understand how their backgrounds and experiences intersected with data analysis [22].

NE’s experiences as a caregiver, woman of color, and health equity researcher contributed to how she interacted with and analyzed the interview data. Having conducted interviews and built a relationship with interviewees, she felt a personal connection to the data and worked to ensure that the invaluable extent of caregiver support in the context of HF was thoroughly reflected in the analysis. Her identities also drove her to achieve representation of diverse racial and ethnic groups within the study sample and balance the representation of all participants within the analysis while also centering the stories, experiences, and suggestions of those who are often underrepresented in health and caregiving research.

CB’s social positionality as a young adult, first-generation Canadian, and member of an ethnocultural minority group largely influenced her ability to empathize with, and understand, the caregiver experience. Although her deeply rooted and culturally embedded moral values surrounding shared familial care allowed her to relate to the motivations behind providing care to a loved one, CB’s lack of firsthand experience in the caregiving space posed a unique challenge to understanding the true extent of daily caregiving and its unique physical, mental, and emotional demands. As an interviewer, CB found that having vulnerable conversations with the participants allowed her to subconsciously build a personal connection and empathize more effectively with the participants and their lived experiences. The personal bond, combined with the emotional recollection experienced by CB, led to unique opinions and perspectives while conducting data analysis.

KY is a second-generation Canadian settler and a woman of color. She has acted as a caregiver, translator, and health care intermediary for multiple family members with chronic illnesses. During the analysis, these identities and labels led her to be conscious and emotionally responsive to how her experiences intersected with each participant’s caregiving story, especially regarding the cultural context of caregiving. She did not complete any interviews during the data collection phase and was only familiar with the participants to the extent to which they provided personal information in each interview transcript.

KG’s positionality as a second-generation Canadian and a woman of color informed her analysis. Although she has not acted directly as a caregiver, her experiences with and observations of the challenges of navigating health care and digital health tools supported her understanding and connections to the participants. Her experiences with equity-focused digital health research and university coursework in the social determinants of health also led to heightened attention to the limitations of technology in the context of complex health systems. She did not conduct interviews during data collection but contributed to the interpretive analysis of transcripts.

### Sample

We obtained a convenience sample of adult ICs (aged ≥18 years) of patients enrolled in the Medly HF management program at UHN. First, the research coordinators reviewed the Medly patient list and reached out to the patient care team for additional screening to identify those who had or may have had ICs engaged in their care. An email was then sent to the Medly patients by the research coordinators, introducing the study and asking for a referral to connect with the IC. A total of 255 patients were approached. All ICs referred by their patient partners expressing interest in being interviewed by the research team were eligible to participate in this research.

### Data Collection

#### Overview

We conducted 20 semistructured individual interviews with a group of ICs across various age groups, ethnic backgrounds, relationship types, and additional social dimensions, as demonstrated in the *Results* section. Our sampling strategy was guided by the exploratory nature of our research aims, pragmatic judgment, and the concept of *information power*, which suggests that the sufficiency of the sample size can be determined based on the pertinence and salience of the information shared by participants [23,24]. In the approach described in the following sections, we aimed to identify common patterns of beliefs and behaviors from participant interviews that were relevant to our research questions. These patterns painted a rich and complex picture of their experiences of caregiving for patients living with HF and how these experiences intersected and interacted with their use of Medly. Demographic information was collected from all participants on age, gender, language, ethnicity, education, living arrangement, area of residence, marital status, employment status, and annual household income. Questionnaires were completed electronically using the REDCap (Research Electronic Data Capture; Vanderbilt University) tool hosted at UHN [25,26]. Demographics are described thoroughly in the *Results* section.

#### Interviews

Using a semistructured interview guide (Multimedia Appendix 1), interviewers NE and CB guided caregivers through questions that explored three main themes: (1) caregivers’ relationship with their care recipient and their experience with caregiving, (2) the caregiver’s role in and views on the Medly program, and (3) opportunities to improve the Medly experience to further support the dyad. Caregivers were asked to reflect on the rewards and challenges of the caregiving experience and provide insights into how they would define a better future. Interviewers guided caregivers through discussions about their personal goals and the barriers they faced in achieving them.

Owing to the COVID-19 pandemic restrictions, all interviews were conducted virtually, either using Microsoft Teams or over the phone. The interviews were audio recorded, and a professional service transcribed the recordings. As expected, the virtual setting of the research posed unique challenges, including establishing rapport with participants and addressing technical issues. Participants chose to conduct their interviews over a video call or over the phone (audio only) based on personal preference. Although phone calls were more accessible to the participants, it was more challenging to build rapport using this modality. Rapport was more effectively built during video call interviews because of the ability to use body language to communicate and create a more human-centered and less formal research environment. To address the nuances of virtual meetings and their associated challenges, our team encouraged participants to complete their sessions through video calls and started all interviews with rapport-building conversations to foster a safe space.

In both phone and video modalities, participants often encountered technical frustrations with electronic consent methods and technical issues associated with navigating Microsoft Teams. To mitigate these frustrations, our team developed a step-by-step guide on how to navigate the teleconferencing tool, which was distributed to the participants via email before their session. For those who continued to experience technical issues, a study team member was able to successfully troubleshot and provide technical support via phone.

#### Codebook Development

The preliminary codebook was developed by 3 team members (NE, CB, and KG) using data from the first 6 interviews based on IC activities outlined by Buck et al [16] and the initial themes identified through familiarization with the transcripts. Specifically, the first 3 interviews were used to conduct a preliminary group listening activity with authors NE, CB, RL, and QP to discuss key ideas, thoughts, and potential feature suggestions for the family-centered model of dyadic digital health. NE, CB, and KG used the remaining 3 interviews to perform a preliminary analysis using the initial codes, with each transcript coded by 2 research team members. Iterative refinement of the codebook was continued with the aim of condensing existing codes, removing ambiguity, and establishing consensus in the codes. This process yielded the final version of the codebook for use in formal data analysis.

### Data Analysis

#### Transcript Coding

We conducted a thematic content analysis of the interview data [27]. A total of 4 research team members (NE, CB, KY, and KG) used the NVivo (QSR International) software to code all the transcripts individually. First, the coders independently read each transcript to familiarize themselves with the data. The transcripts were then assigned and coded independently, while the coding team met weekly to discuss questions, resolve differences, and refine the codebook through an iterative review of the themes derived from additional interviews. The team maintained reflexivity journals to reflect on how their experiences, background, and positionality affected and intersected with their coding work. Themes were first derived deductively based on the structure of the interview guide and using HF typologies, as defined by Buck et al [16]. We then inductively refined the analysis to incorporate the identified themes, borrowing from the approach outlined by Braun and Clarke [27]. Finally, the themes identified from the transcript coding were recategorized based on concepts from the van Houtven et al [18] framework, which provides a structure for evaluating caregiver interventions.

We used a multitude of practices and methods to ensure the quality of our study, as described in the 8 *big-tent* criteria for high-quality qualitative research conceptualized by Tracy et al [20]. Specifically, we aimed to build methodological rigor through the use of relevant frameworks, rich descriptions of the context and sample from which data were collected, and the use of appropriate and theoretically informed data collection and analytical methods. Sincerity, another key criterion, is spoken to in our description of our reflexivity processes and the filter of our positionality statements; that is, we seek to uncover and make ourselves and our readers aware of our motivations for pursuing this work and honestly put forward the biases we may have held in the process of data collection and analysis.

#### Statistical Analyses

Descriptive statistics are reported for all demographic data collected from the study sample. The number and percentage of participants who responded to each question, as well as the mean age of participants (reported with the SD), were calculated and are presented in the *Results* section. Missing responses were removed from the total count when calculating the percentage and mean age and were noted where applicable.

### Ethics Approval

All recruitment and data collection activities were approved by the UHN Research Ethics Board (REB 20-5238).

## Results

### Overview

A total of 20 ICs were interviewed in this study. The average age of the participants was 63 (SD 7.5) years, and most identified as women (17/20, 85%) and White (14/20, 70%). However, our sample also included 25% (5/20) of participants who spoke English as a second language and 25% (5/20) who identified with diverse racial and ethnic groups. We acknowledge that this sample experienced favorable socioeconomic conditions, with 65% (13/20) of participants educated above the high school level and 40% (8/20) earning an annual household income of >CAD $100,000 (US $77,170). Most caregivers in our sample were assessed to belong to a collaborative dyad typology (10/20, 50%; 5/20, 25% patient-oriented; 5/20, 25% caregiver-oriented). Approximately 55% (11/20) noted that they were wives caring for their husbands who lived with HF. For a detailed breakdown of the demographic information, refer to Table 1.

Interviewers and participants were able to connect through shared experiences and the ease of conversation about the interview topics. Our qualitative analysis yielded two salient themes pertaining to (1) IC experiences with HF caregiving activities and (2) the role of technology in facilitating HF caregiving activities within a dyad. Consistent with the study by van Houtven et al [18], these themes encompassed the amount of caregiving, psychological skills, support-seeking activities, and domains of clinical knowledge.

**Table 1:** Demographic characteristics of informal caregiver participants (N=20).

Individual-level variable			Value
**Age^a^ (years)**			
	Values, n (%)	19 (95)	
	Values, mean (SD)	63 (7.5)	
**Gender^b^, n (%)**			
	Woman	17 (85)	
	Man	3 (15)	
**Patient-caregiver dyad typology, n (%)**			
	Patient-oriented	5 (25)	
	Caregiver-oriented	5 (25)	
	Collaborative	10 (50)	
**Care relationship, n (%)**			
	Wife caring for husband	11 (55)	
	Husband caring for wife	3 (15)	
	Daughter caring for parent	4 (20)	
	Mother caring for daughter	1 (5)	
	Friend caring for friend	1 (5)	
English as a second language, n (%)			5 (25)
**Race or ethnicity, n (%)**			
	South Asian	1 (5)	
	Black: African or Caribbean	2 (10)	
	Indigenous or Aboriginal	1 (5)	
	Latin American	1 (5)	
	White: European or North American	14 (70)	
	Other(s)	1 (5)	
**Highest level of education, n (%)**			
	High school diploma	4 (20)	
	College trade or technical diploma	5 (25)	
	University undergraduate degree (eg, BA or BEng)	6 (30)	
	University professional designation (eg, MD or MBA)	2 (10)	
	Other(s)	3 (15)	
**Employment status, n (%)**			
	Retired	9 (45)	
	Unemployed	1 (5)	
	Working full-time or part-time	8 (40)	
	Other	2 (5)	
**Annual household income (CAD $ [US $])^a^, n (%)**			
	<49,999 (<38,537)	2 (10)	
	50,000–100,000 (38,538–77,075)	8 (40)	
	>100,000 (>77,075)	8 (40)	

^a^Indicates missing responses.

^b^Participants were provided with the option to select “non-binary” or “prefer not to answer,” of which no selections were made.

### IC Experiences With HF Caregiving Activities

#### Overview

A critical factor that inherently influences the amount of caregiving that an IC engages in is the nature of their dyadic typology. Our sample included patient-oriented (patient performs most tasks), collaborative (patient and caregiver share care tasks), and caregiver-oriented (caregiver performs most tasks) dyad typologies, each conferring distinct caregiving requirements. Specifically, a caregiver-oriented dyad often provides a greater amount of caregiving as the patient requires more support with activities of daily living along with improving and maintaining self-care. In addition, compared with patient-oriented and collaborative dyads, patients in caregiver-oriented relationships are often much more dependent on their caregivers:

I basically do all of it. I make sure her appointments are done; I take her to all her appointments...I do all her medications, I fill her pill boxes, I call her on the phone when it’s time to take her medications...I do all her vitals in the morning, I do her blood pressure, her heart rate, her temperature twice a day. I look after anything health.

C05, caregiver-oriented

In contrast, patients in patient-oriented dyads were able to maintain their independence with most of their self-care tasks while receiving support from the caregiver when needed:

The other ways that I help him is I try to encourage him to go for walks or to, like, be more—a little bit more active...But other than that, he’s pretty self-sufficient in doing his own [care]—he cooks and [eats] the right things.

C08, patient-oriented

The level of involvement of ICs in the care can also depend on the extent of the barriers that the care recipient experiences in interacting with the health care system (eg, visual impairments, cognitive implications, and language barriers) and the desire to provide their own care. In some instances, caregivers provide more care and are able to cope well with this arrangement, whereas others provide less care than desired because of the patient’s preference to be self-sufficient and manage their own care:

My husband wants to be engaged. If he feels, he always feels left out. And his favourite comment to me is, “it’s my body. It’s my medical condition, and everyone is making decisions without my being involved.” My husband is colourblind, so he can’t see. He can’t participate. He couldn’t input the information [into Medly], because when it comes up [it’s in] red...So this is why I’m managing it.

C09, caregiver-oriented

The amount of caregiving influences the overall IC experience as dyads continually adapt and navigate effective strategies to manage living with a diagnosis of HF within their unique circumstances. Participants from all typologies shared their caregiving experiences, the uncertainty embedded in the role, and the specific challenges they faced around (1) living with pervasive unpredictability and lack of control, (2) support-seeking behaviors to mediate the degree of IC burden, and (3) the high-stakes learning curve associated with HF diagnosis and management. Each of these is expanded in the following sections.

#### Living With Pervasive Unpredictability and Lack of Control

Across all dyadic typologies, the sudden onset and unpredictable nature of an HF diagnosis often leave caregivers feeling helpless and reporting a lack of control over the patient’s HF prognosis. In addition, the severity and impact of HF exacerbation events throughout the HF journey can be traumatic:

It was a scary moment having her collapse in my arms that day and then seeing them work on her for over half an hour trying to get her to come back to life. That was really scary. The doctor said to her that she had a 3% chance of surviving and the fact that she didn’t have any brain damage or organ damage was another miracle.

C17, collaborative

For me as a caregiver, at times it can be frustrating—I guess because of the fear. The fear of what if this [cardiac arrest] happens again? I certainly don’t want it to happen again, but living with heart failure, it’s...you just don’t know.

C10, patient-oriented

The challenge of uncertainty makes every day unique and difficult to anticipate for caregivers:

We don’t know what to expect day-to-day. It’s not like we can say “Well it’s been a good week, you know, he’ll be fine tomorrow,” because tomorrow he may not be fine...You can’t make long-range plans.

C13, collaborative

The IC’s sense of self is often affected by sudden and unexpected changes in the care recipient’s health arising from an HF diagnosis or exacerbation event. The lack of time to grieve and process the change results in caregivers feeling overwhelmed and helpless:

It took a couple of years to get over [my husband’s cardiac arrest]. You’re doing CPR on your husband. So it was just the trauma of going through that experience. And then when he did come home, seven weeks later, he’s so weak. He ended up collapsing on the floor. It [wasn’t until] a couple years later we had gone to the ICD Clinic and one of the doctors there, he said, “Well how did you feel?” That was the first time someone had asked me how I had felt through that experience. So I was ready to break down in tears at that time.

C10, patient-oriented

These rapid changes also affect the dynamic within the dyad and may confer additional stress on the IC as they work to balance caregiving for patients engaging in self-care activities. There is often guilt associated with the latter, as the caregiver struggles to take time away from the caregiving role to focus on their own physical health and well-being. These intersecting feelings of self-neglect and grief are especially amplified in caregiver-oriented and collaborative dyads, as caregivers are required to adjust their lifestyle and activities to a larger degree to accommodate the care recipient’s needs:

Unfortunately, what happens is as one partner becomes less active, the second person also becomes less active because you feel guilty. For example, we could go out for a walk, which is recommended. But obviously, my husband doesn’t feel the same way every day...So if he can’t go out, I’m not going to go out and leave him.

C09, caregiver-oriented

I don’t do what I used to do at all. And I don’t want to sound selfish or anything but it seems like anything I need or I want to do is always second. Everything is always based around him, which is how it should be, but sometimes emotionally that gets to you where you just kind of, like, want to run away and have a date with yourself sort of thing.

C18, collaborative

The lack of control that the caregiver has over the patient’s health behaviors, reactions, and perceptions may contribute to additional stress on the IC and add further strain on the dyad’s relationship. The degree of congruence between the IC and care recipient regarding expectations of how to engage in care tasks and who is responsible for different tasks affects their appraisal of the caregiving experience. A dyad with similar expectations generally predicts a more sanguine approach to caregiving, which may reduce the psychological toll of the experience. However, discrepant views on dyadic expectations and the patient’s care plan may result in additional stress for the IC, as the dyad shifts between being independently oriented, as in caregivers providing support, and a collaborative approach to care where care recipients are empowered and their independence is fostered:

We have this back and forth because he’ll just take [his Medly readings] and leave [the paper that has the readings written on them] and then expects me to look for it and put it in [the Medly app]. And I say, “No. You [do it]’ because I just like him to be a little bit on the up and up, like to being responsible in doing that.”

C08, patient-oriented

The psychological toll associated with discrepant views is especially heightened for caregivers in caregiver-oriented and collaborative dyads, where the caregiver is more heavily involved in the patient’s care.

#### Support-Seeking Behavior to Mediate the Degree of IC Burden

Outlets that diffused stress or acted to alleviate caregiver burden were crucial components of caregivers’ support systems and, by extension, the dyad as a whole. Outlets included family, friends, health care providers, financial stability, respite hours, and disease management tools among others.

Experiencing a high level of uncertainty in the caregiving journey without the presence of a support system decreased the ICs’ ability to provide quality care and increased caregiver burden and burnout. This was most salient for caregiver-oriented dyads, whereby ICs were significantly relied on for support from their care recipients.

We don’t have extended family here. Or friends. So it’s just my family and that’s it. Him and I and my son and that’s it...I have been to the point where I can’t take it anymore, overloaded, overwhelmed. And it affects me a lot.

C20, caregiver-oriented

Conversely, the dyadic relationship typically improved when caregivers had the ability to know when to seek support and had support available to diffuse the stress and responsibility they experienced. In this way, support systems aided the whole dyad; the care recipient continued to receive quality care, and the caregiver was able to receive respite from caregiving responsibilities:

So what I personally do is...one of my sisters...comes down and spends two nights with me every week...And it gives me a bit of relief, it gives me a bit of socializing that’s just for me, where I can go outside and be in the bush for an hour and she stays in the house [with the patient]...So she’s like a second me when she’s here...And if it wasn’t for her doing that I’m not sure what state I would be.

C18, collaborative

Finding appropriate support systems enables dyads to find a balance and simultaneously contributes to improved self-efficacy and resiliency among caregivers. Furthermore, the opportunity to share experiences with others who have had similar HF experiences can provide an invaluable source of support. It provides caregivers with reassurance, guidance, and a look into their future:

I was completely lost [caregiving for my daughter with HF]. And out of the blue, a friend of mine revealed to me that she had lived with heart failure for 20 years...She gave me so much hope, so much courage. That call came to me at a time when I didn’t know what to expect. She used to call [my daughter to] ask her how she was feeling and give her own tips of how she had coped. So I’ve been lucky to have supportive friends as well as family. And that’s what has really kept me going as a caregiver. And as a mother. It’s not just a caregiver hired, but a caregiver because she’s your child.

C21, collaborative

#### The High-Stakes Learning Curve Associated With HF Diagnosis and Management

Caregivers described a high degree of uncertainty in their care experience, often derived from their fundamental lack of HF knowledge and professional training. This lack of knowledge inherently left caregivers feeling less confident in their ability to support their loved one with appropriate symptom management. Although knowledge can be a powerful tool to help reduce uncertainty, caregivers also shared that finding high-quality, trustworthy resources is difficult. Navigating where to turn and what to trust is complex, especially when sourcing evidence-based and up-to-date guidance on HF management. Self-doubt weighs heavily on these caregivers. The yearning for practical knowledge and greater direction on how to support their care recipient is a common sentiment felt among ICs who have more hands-on experience and those who are responsible for several care tasks. There are numerous opportunities to doubt their own subjective assessment:

He had an infection. But what’s normal? What isn’t? And we’ve heard every individual is different, but even to have the ability for me as a caregiver to say oh, there seems to be more discharge at the driveline site. What are you looking for? Or what would be a red flag for that?

C14, collaborative

Sometimes I’ll just say, “Ok, well just lie down.” But who knows if that’s the right thing to do.

C06, collaborative

The only thing in the beginning was the medication. Because all the words are so foreign to me. I just take notes and it’s up to him to take the medication...I was afraid that I would mess up on that...miss a time when he was supposed to have it.

C13, collaborative

Moreover, the frequency of HF exacerbations and the need for ongoing supportive care to prevent and manage them leaves ICs with a sharp, high-stakes learning curve as they navigate and manage the HF journey:

I have to understand or accept that fact that this is who he is right now, this is what he can do right now, this is what I can expect from him, no more. It’s been a learning curve for me.

C20, caregiver-oriented

### The Role of Technology in Facilitating HF Caregiving Activities Within a Dyad

#### Overview

Although Medly was designed to be used independently by the patient, many ICs took over the Medly user role on their behalf to enable participation in the program during the COVID-19 pandemic. Other dyads collaboratively engaged in the use of Medly to provide supportive shared care. Although caregivers tended to trust the medical guidance provided by Medly as it came from a trusted institution, this was not always the case for some care recipients. Dealing with nuanced perceptions of trust within the dyad posed unique challenges for the caregiver as they attempted to address the patient’s hesitancy. As Medly is a prescription-based DTx, pre-existing relationships with clinical staff and the patient’s health care team also contributed to a sense of trust within the program. Building on this foundation of trust fostered an environment in which Medly could serve as both a DTx and a component of the IC’s support system. The remainder of this section outlines the dyads’ experiences and the potential role of technology in supporting ICs by (1) reducing uncertainty and providing peace of mind, (2) acting as a *member* of the dyad’s support system, and (3) mitigating the dyad’s clinical knowledge requirements.

#### Technology May Reduce Uncertainty and Provide Peace of Mind

Earlier, we noted the magnitude, impact, and weight that uncertainty imparted to ICs. Medly’s regular check-ins and accessible connection to clinical staff provide a release valve to diffuse stress associated with the care recipient’s unknown disease progression and acute status. In this way, technology can reduce uncertainty by providing dyads with personalized directions on what they need to do to best manage the patient’s condition on a day-to-day basis:

When I put in the data if there’s anything a little bit off with it, it’ll tell me, this is too high, this is too low, this is a concern. I like that. Because then I know that it’ll tell me if I should make him redo the measurements in the afternoon.

C08, patient-oriented

The quick response to the information that’s inputted is fabulous. It tells me whether we’re on track or we’re not on track. And then I could modify on a day to day basis...it also provides me [with] a trend line...if I want to look at the last week and see what the trend is, I can see what we’re doing.

C09, caregiver-oriented

The ability to connect with experts through Medly also provides invaluable peace of mind to the dyad:

Sometimes they’ll just notice that [her weight is] starting to creep up and they’ll [advise], “OK you should take that drug and go get the blood tests done.” And so it’s nice knowing that somebody is keeping an eye out.

C17, collaborative

Through Medly, the care recipient’s health is monitored on an ongoing basis where they would otherwise be left alone to manage the condition without expert oversight outside of synchronous touchpoints with the clinical team. This peace of mind has a profound impact on relieving a significant proportion of caregiver burden:

I mean she came home from the hospital...her procedure was a bit critical in the hospital...Just being able to come home and hook [Medly] up, it just really gave us a good sense of security and comfort just knowing that she was being monitored on the other end as well.

C06, collaborative

#### Technology Can Act as a Member of the Dyad’s Support System

Medly is a key element of the dyad’s support system for 3 reasons: the support provided by Medly is instantaneous; there is a direct point of contact with clinic staff available through the service during business hours; and it facilitates symptom tracking, which reduces the dyad’s burden of monitoring:

I’m not [asking] every morning, “Well what is it? What are your numbers today?” I might do that for a week or so and then it wears off. I know that if I haven’t asked what the numbers are...the coordinator’s going to call me and say this has happened and this is what you need to do.

C13, collaborative

I kind of feel like [Medly]’s almost taking that role of being kind of her caregiver. I’m here for everything else if she ever needed anything, but I almost feel like it’s kind of my mom’s caregiver to be quite honest. It’s kind of taken a bit of that off of me.

C24, patient-oriented

Medly’s daily input requirements and feedback mechanisms often serve as discussion prompts between the caregiver and care recipient, thus enabling better communication within the dyad:

It’s also become like there’s something in common for both of us to discuss, to analyze, to find out how he feels, is there any difference today and look at the values every day when he takes his reading, are we within the parameters they have given us. And I know that it’s not just the value, it also depends on how he feels so there’s a conversation always going around that.

C11, collaborative

The ICs appreciated Medly for its ability to provide continuity of care while taking over some of the organizational skills that caregiving typically requires, such as providing medication prompts during exacerbation events and medical care in emergencies.

#### Technology Can Mitigate the Dyad’s Clinical Knowledge Requirements

Empowering patients and formalizing the off-label use of Medly by caregivers can facilitate positive dyadic changes. The knowledge and medical guidance provided by Medly enable a greater understanding of the patient’s daily HF status within the dyad:

Before being on Medly, he wasn’t even sure if he was feeling well or wasn’t. So...having some kind of ability to punch in numbers and have an expert look at them and know if they’re good or not good was just crucial.

C14, collaborative

On the other side of the app there are human beings who are very well trained and who are specialized in heart failure. I think this program is amazing because of the knowledge base that it has.

C04, caregiver-oriented

The constant feedback mechanism associated with Medly’s daily check-in prompts facilitates greater awareness of the patient’s HF status between care recipients and caregivers:

It’s really been a very handy tool and it’s provided him with the ability to care for himself too. When he’s not feeling well, he [uses] this tool as a means of keeping connected with health professionals...He’s using it every day, recording his symptoms. It helps him keep in line with, “OK. I need to watch my diet, my fluids, take my medication, check my blood pressure, check my weight,” so it’s been a very useful tool for him. For me too, because then I know that he’s taking check of what’s going on with how he’s feeling.

C10, patient-oriented

Technology can empower ICs to feel more competent and confident in their caregiving abilities by providing a medium to access evidence-based information and actionable feedback for the patient’s dynamic HF status. In this way, technology can act as a dyadic self-efficacy tool for HF management.

## Discussion

### Principal Findings

Underlying many of the experiences and anecdotes shared by ICs of patients with HF was the stress of uncertainty regarding a patient’s future disease status. HF is a progressive chronic disease that naturally worsens over time. However, progression is nonlinear, and HF exacerbation events cannot always be predicted. This uncertainty is more difficult to cope with if a caregiver within a dyad perceives that they lack the knowledge or support to effectively or adequately care for their care recipient.

Although several IC-facing health technologies exist within the private sector, very few have been formally tested through traditional research. Of the existing technologies that have been evaluated, many have shown to be efficacious in their ability to improve primary and chronic care access, especially in older ethnic adult populations [26]. A systematic review of telehealth interventions built to support ICs showed significant benefits across dyads. The benefits included improved psychological health, confidence, knowledge, patient management, communication with providers, and physical health [28]. Similarly, an experimental study that engaged patients living with HF and family caregivers in an interactive voice response intervention demonstrated the positive effects of dyadic engagement, which resulted in enhanced patient medication adherence, caregiver communication, and quality of life among patients while decreasing the likelihood of HF exacerbations [29].

Therefore, there is an opportunity for technology to better support improved outcomes in HF dyads. For patients experiencing cognitive decline, language barriers, visual impairments or possessing minimal digital literacy, caregivers often stepped in as Medly proxy users on the patient’s behalf. In contrast, caregivers in dyads with patients who had few accessibility challenges generally took on a more supportive role and did not directly interact with Medly. Our results suggest that ICs in caregiver-oriented dyads may be more likely to act as proxy users of digital health interventions. With their involvement, patients can overcome this digital divide and adopt digital health interventions. In our study, the ICs across all 3 dyad typologies expressed myriad benefits, from reduced care burden and improved family functioning to an improved sense of security in the care provided to their patient and improved ability to cope with caregiving-related stress. Our findings are aligned with other studies on digital health interventions that formally engage ICs in shared dyadic care and demonstrated improved outcomes; however, this avenue of research remains nascent in application and practice [28].

Our findings further identified a need to focus on improving the dyadic experience as a whole by building IC functionality into digital health self-management interventions. An avenue to address this need would be to formalize the IC role within Medly to address dyadic needs across the spectrum of caregiver engagement (ie, primary or secondary ICs). This approach was supported by the scoping review by Park et al [29] assessing the quality of family-targeted caregiver apps, which highlighted that appraised apps commonly neglect caregiver needs by focusing solely on improving the patient’s provision of care.

Although Medly was designed to be patient facing, we have presented scenarios in which Medly was used off label by the IC as a proxy. The dyadic nature of HF caregiving was not initially incorporated into Medly as a design consideration, and we acknowledge that targeted caregiver technology may be more beneficial for certain dyadic typologies. Indeed, feedback from caregivers indicates that the addition of a caregiver account to Medly would be beneficial in diffusing the caregiving burden across the patient’s circle of care in times of decline in the health of the patient when the demands of caregiving typically rise. However, the desire for caregiving functionality varied across dyadic typologies. ICs in caregiver-oriented typologies saw less added value in a proxy account as they already used Medly on behalf of the patient through the primary patient account. For them, new caregiver functionality would have limited the utility without additional features that extended beyond the current Medly offering (eg, caregiver mental health support). In contrast, caregivers in collaborative and patient-oriented dyads expressed greater interest in having a caregiver account so that they could remain informed of the care recipient’s health status without having to ask the patient directly:

It would be great for me to be able to jump on the app and just have a look and see, well, how many pillows did she sleep with last night? Is she having a hard time doing the stairs, that kind of thing...I think she doesn’t want to worry me.

C24, patient-oriented

Considering the role technology can play in providing peace of mind to dyads, design considerations for accessibility must be prioritized. Inclusive design should be the standard for digital health as it significantly influences the uptake of innovations such as Medly. Failing to proactively consider and design for how older adults and their ICs might use technology for HF management might prevent dyads from meeting their care goals. The ICs in our study informed that their patient partners relied on them to engage with the Medly app because of visual impairments that made it difficult for them to use the app independently. This feedback motivates the need to implement design changes and ensure that the Medly app provides sufficient color contrast, audio commands, font size options, and responsive screen compatibility:

My husband is colorblind. So if...there’s an alert that comes out in red, for example, he could not see that it’s red, and there’s a problem. There are programs that you can actually speak and the computer will type it up for you or send a message...I’m wondering if they [could] engage Medly [and it would say], “patient X, what is your weight today? What is your blood pressure today?” and you can verbally respond? And the output would be verbal as well. “Your blood pressure is fine today. Your weight is fine today or your weight is high today, please take two additional ABC medications.”

C09, caregiver-oriented

Viewing the results, it’s really hard to look at things historically because the screen is so small. It’s hard to figure out where he was at certain points because you can’t see it on such a small screen.

C03, caregiver-oriented

In cases of patient mistrust of the health care system or technology, caregivers may be required to step in and facilitate acceptance of and access to digital health. Such mistrust is especially important to consider in patients belonging to communities that have historically experienced, and continue to experience, challenging and traumatic interactions with these systems, including older adults and those from ethnocultural minority communities. Cultural safety and language accessibility are critical to support the management of chronic conditions that disproportionately affect ethnocultural minority communities [28]. Promoting inclusivity in digital health through equitable design and implementation strategies may simultaneously improve the uptake of remote HF management using technology while mitigating the burden of caregiving on ICs in dyads and enhancing care recipients’ autonomy.

### Strengths and Limitations

Although research on IC engagement with DTx is scarce and still emerging, our research team was uniquely positioned and able to explore the experience of ICs with partners enrolled in the Medly program at the UHN. This position allowed us to elucidate the complexity of care needs within the dyadic unit as they sought to comanage HF. These perspectives have not been well represented in prior research, and the portrayal of the experiences of ICs within their HF dyads is a strength of our study. However, this study has several limitations. As a result of our focus on ICs, patient perspectives were not directly assessed as part of this initial phase of our exploratory research. Gathering insights from both entities within the dyadic unit would have provided more holistic insights and is planned for the future phases of this study. In addition, the research team originally sought to explore novel ways of supporting older ethnic adults with limited English proficiency and their ICs in the use of DTx. However, we recognize that most of our IC sample was skewed toward those who self-identified as White (14/20, 70%). Further research on the barriers to accessing digital health within ethnocultural minority populations, which integrates intentional, culturally safe recruitment strategies, is necessary to warrant a more diverse and generalizable sample. In response to this limitation, detailed descriptions of the study results were provided to contextualize the study findings. More generally, future work at the intersection of dyadic HF management and technology use is warranted with other digital health platforms to further evaluate the generalizability of our findings.

### Conclusions

We observed the value of an off-label implementation of the Medly HF DTx in improving the dyadic HF management experience. We believe there is a need to formally and intentionally expand HF technologies to include dyadic needs and goals, considering both the caregiver and the care recipient. When planning technology design and the value of technology to support care dyads in managing chronic conditions, we suggest defining 3 opportunities for support. The first is to identify how technology may be leveraged to increase psychological bandwidth by reducing uncertainty and providing peace of mind. We found that actionable feedback was highly desired by both partners. The second is to develop technology that can serve as a member of the dyad’s support system. In our experience, automated prompts for patients to take measurements can mimic the support typically provided by ICs and ease their workload. The third is to consider the ways in which technology can mitigate the dyad’s clinical knowledge requirements and learning curves. Our approach was to have real-time actionable feedback paired with a human-in-the-loop, nurse-led model of care. Through a shared model of dyadic care that supports the role of the patient in their own HF management, includes ICs to expand and enhance the patient’s capacity to care, and acknowledges the needs of ICs to care for themselves, we anticipate improved outcomes for both partners.

## Appendix

Multimedia Appendix 1 Interview guide.

## Abbreviations

DTx: digital therapeutic
HF: heart failure
IC: informal caregiver
REDCap: Research Electronic Data Capture
UHN: University Health Network
