# Supplementary material for: The Value of Technology to Support Dyadic Caregiving for Individuals Living With Heart Failure: Qualitative Descriptive Study
Source: J Med Internet Res. 2022 Sep 7;24(9):e40108. doi: 10.2196/40108 (PMC9494221; doi:10.2196/40108)
Supplement: Multimedia Appendix 1 [file jmir_v24i9e40108_app1.docx]

## **Multimedia Appendix 1 -** Semi-structured interview guide

*The intent of these interviews with caregivers is to adapt Medly to better involve and support caregivers. As you may know, Medly is a heart failure telemonitoring program where patients are able to enter their daily readings (blood pressure, weight, symptoms) and receive alerts and instructions based on the results of their readings to help them self-manage their health. For many of our patients, they use the app by themselves and manage their health independently, but more of our patients in the Medly program are enrolling in the program hand in hand with their caregiver, for varying reasons, making caregivers involved in using the app to different extents (not at all, viewing readings, tech support, entering data etc.). Because of this, we are aiming to create a caregiver account in Medly where caregivers would be able to see the daily Medly data entries of the person they provide care to in addition to other features like being able to input data on behalf of the patient, and chat directly with the nurse coordinator.*

*So keeping this in mind - today, I want to learn about your experience as a caregiver as well as your goals. Throughout the interview, I encourage you to think about how you would like your future as a caregiver to look like so we can find ways for Medly to better support you in achieving your goals.*

**Theme 1: Exploring the patient-caregiver relationship and the caregiving role and experience**

To start off, I want to learn about what your experience being a caregiver is like.

1. Can you tell me about your relationship with the person you provide care to?
2. How are you involved in their care? What are some ways that you help them with their health?

*Probes:*

- How long have you been providing care for them, and how has your caregiving changed over the years?
  - - What are some things you wish you knew when you first started providing care that you know now?
  - How did you come to be their caregiver?
    - What role did your culture play in the decision to take on caregiving responsibilities? We define culture as the shared values, beliefs, traditions, languages, religions, behaviours, and other characteristics that are handed down from one generation to the next.
    - What motivates you to continue caring for your [person]?
  - What caregiving responsibilities do you perform? (i.e. medication/ diet/ exercise reminders, prescriptions, appointments, emotional support)

1. What would you say is the main health management goal that you are looking to support them with? (e.g. comfort, quality of life, HF symptoms)
2. What are some challenges you face while providing care? (*take notes*)

*Probes:*

- What are some tools, resources, or strategies that help you to overcome these challenges?

1. What are some rewarding moments you experience while providing care?
2. What effect has caregiving had on your life?

*Probes:*

- - What has changed in your life since you became a caregiver?

1. Is there anything else you would like to share about your experience as a caregiver?

**Theme 2: Understanding the caregiver’s experience with Medly**

Your [person] is currently enrolled in the Medly program to manage their heart failure.

1. How do they use Medly?
2. What is your experience with Medly? How do you currently interact with Medly in your caregiving role?

*Probes if caregiver uses Medly:*

- - What features of Medly help you in your caregiving role? What features of Medly make it challenging for you to provide care?
  - How did you and your [person] decide on your current roles for using Medly?
  - Are there any privacy challenges or issues that come up by using the Medly program as a caregiver?
    - How do you feel about being able to see all of your [person’s] health information that goes into the app? How do they feel about it?

*Probes if caregiver does not use Medly:*

- - How do you feel about the Medly program?
  - How does Medly impact your role as a caregiver?
    - What aspects of caregiving does it facilitate?
    - What aspects of caregiving does it make more difficult?
  - What concerns do you have about the program?
  - What do you think are the benefits of using it?

1. What are your thoughts on a caregiver account? Again, this would allow caregivers to see the daily Medly data entries of the person they provide care to along with access to other features like being able to input data on behalf of the patient, and communicate directly with the nurse coordinator.

*Probes:*

- - Are there any other ways that you would like to be able to use the app as a caregiver?

**Theme 3: Goals and Opportunities**

I understand that caregiving is one aspect of your life, but it is not the only aspect. In this section, I want to learn about your personal goals. I also want us to look towards a better future to brainstorm together on what could be done to better support you in achieving those goals.

1. I want to learn about the goals you have related to different aspects of your life, such as physical and mental health, education, employment, finances, and relationships. What would you say are the things you want to accomplish (now and in the future) in your personal life? As a caregiver?

*Probes:*

- - We’ve heard that many caregivers have goals like getting more sleep, fitness, coping better, having a life outside of their care situation, and better manging the financial demands of caregiving. Do you relate to any of these?

1. It sounds like your main goals are [*summarize goals here*]. How would you say your caregiving role affects your capacity to achieve these goals?
2. You mentioned previously that some challenges you experience in your caregiving role are [*summarize challenges from Theme 1 here*]. Thinking of a better future, what would help make those challenges less of an issue?

*Probes:*

- - How could technology help relieve some of these challenges?

1. Thinking about a caregiver account in Medly – what kinds of features in the app might help you accomplish your goals?

*Probes:*

- [*For caregivers who use Medly*] What would you like to be able to do on Medly that you currently can’t do?
- I’m going to walk through some suggestions that we’ve heard from other caregivers, and I want to know what you think of them, and if they would be useful to you in achieving your goals.
  - Educational resources or information that gives you guidance on how to support the patient with their symptoms/alerts (i.e. concrete actions that you can take as a caregiver, questions you could ask them about their change in health, or things you might be able to change in your day to day life to better support them)
  - Training on how to use the app and take readings
  - Option to enter other symptoms or contextual info
  - Medication tracker/prompts
  - Peer support and community feature
  - Mental health support (e.g. prompts to check-in on your mental health)
  - Guidance or tips on how to co-manage health as partners

**Closing**

1. Is there anything else you would like to add about your caregiving experience or about how Medly could better support you as a caregiver?
